# *Ribes nigrum* Leaf Extract Preferentially Inhibits IFN-γ-Mediated Inflammation in HaCaT Keratinocytes

**DOI:** 10.3390/molecules26103044

**Published:** 2021-05-20

**Authors:** Andrea Magnavacca, Stefano Piazza, Anna Cammisa, Marco Fumagalli, Giulia Martinelli, Flavio Giavarini, Enrico Sangiovanni, Mario Dell’Agli

**Affiliations:** 1 Department of Pharmacological and Molecular Sciences, University of Milan, 20133 Milan, Italy; andrea.magnavacca@unimi.it (A.M.); stefano.piazza@unimi.it (S.P.); marco.fumagalli3@unimi.it (M.F.); giulia.martinelli@unimi.it (G.M.); flavio.giavarini@unimi.it (F.G.); mario.dellagli@unimi.it (M.D.); 2 Specialist in Dermatology and Venereology, Corso di Porta Romana 131, 20122 Milan, Italy; acammisa.doc@gmail.com

**Keywords:** *Ribes nigrum*, blackcurrant leaves, skin, inflammation, keratinocytes, IFN-γ, TSLP

## Abstract

*Ribes nigrum* L. (blackcurrant) leaf extracts, due to high levels of flavonols and anthocyanins, have been shown to exhibit beneficial effects in inflammatory diseases. However, whereas their traditional use has been investigated and validated in several models of inflammation and oxidative stress, the possible impact on skin disorders is still largely unknown. The purpose of this work was to elucidate the effects of *R. nigrum* leaf extract (RNLE) on keratinocyte-derived inflammatory mediators, elicited by a Th1 or Th2 cytokine milieu. HaCaT cells were challenged with TNF-α, either alone or in combination with the costimulatory cytokines IFN-γ or IL-4, and the release of proinflammatory cytokines and mediators (IL-8, IL-6, s-ICAM-1, and TSLP) was evaluated. The results showed that RNLE preferentially interferes with IFN-γ signaling, demonstrating only negligible activity on TNF-α or IL-4. This effect was attributed to flavonols, which might also account for the ability of RNLE to impair TNF-α/IL-4-induced TSLP release in a cAMP-independent manner. These results suggest that RNLE could have an antiallergic effect mediated in keratinocytes via mechanisms beyond histamine involvement. In conclusion, the discovery of RNLE preferential activity against IFN-γ-mediated inflammation suggests potential selectivity against Th1 type response and the possible use in Th1 inflammatory diseases.

## 1. Introduction

*Ribes nigrum* L. (blackcurrant) is a deciduous shrub belonging to the family Grossulariaceae and native to the temperate regions of the northern hemisphere. A number of scientific papers have shown the beneficial effects of blackcurrant in inflammatory diseases due to high levels of anthocyanins and proanthocyanidins [1]. Herbal teas made with blackcurrant leaves have traditionally been used to relieve urinary complaints and minor articular pain [2]. Accordingly, the oral administration of the dried leaves, standardized to the flavonoid content (>1.5%, expressed as rutin), has been reported by the ESCOP monographs as adjuvant in the treatment of rheumatic conditions [3]. The main flavonoids occurring in blackcurrant leaves belong to the class of flavonols, of which the most mentioned are quercetin and kaempferol glycosides [4]. In addition, proanthocyanidins and phenolic acids may occur as well [5].

The traditional use of blackcurrant leaf extracts rich in polyphenols has been investigated and validated in various models of inflammation [6,7] and oxidative stress [8]; however, the possible impact on skin disorders is still largely unknown. Recently, our research group extensively reviewed the preclinical data on the effect of *Ribes* spp. At the skin level [1]; an ointment containing 1% methanolic *R. nigrum* leaf extract showed wound-healing properties in vivo [8], while the oral administration of a quercitrin-rich hydroalcoholic extract of *Ribes fasciculatum* Maxim. roots attenuated the allergic response in a mouse model of atopic dermatitis (AD) [9].

According to the latest scientific advances, the role played by keratinocytes in the pathogenesis of inflammatory-based skin diseases is crucial. Keratinocytes directly interface with the external environment, taking part in the barrier homeostasis and communicating with the resident immune system. The alteration of these structural and regulatory functions is associated with common skin diseases such as psoriasis and AD (for excellent reviews on the topic, see Kim et al. [10] and Girolomoni et al. [11]). An antimicrobial peptide (cathelicidin or LL-37), released from injured keratinocytes, was recently recognized as the antigen triggering the autoimmune response occurring in psoriatic patients (Th1-mediated), which is characterized by elevated levels of IFN-γ produced by Th1/Th17 lymphocytes [12]. Similarly, cytokines released by damaged epidermis, such as thymic stromal lymphopoietin (TSLP) and IL-33, may trigger the pruritogenic and humoral response in AD patients (Th2-mediated), mediated by elevated levels of IL-4, IgE, and histamine (see the review by Corren and Ziegler [13]). Notably, psoriasis and AD are commonly referred to as prototypes of Th1 and Th2 inflammatory diseases, respectively. Nevertheless, mediators from both types of inflammatory response are involved in their pathogenesis.

Several in vitro studies have elucidated, at least in part, the way in which keratinocytes may respond to innate immunity (TNF-α, IL-1β), as well as Th1 (e.g., IFN-γ, IL-17) and Th2 (e.g., IL-4) cytokines. Firstly, they are able to amplify the inflammatory process in response to innate immunity cytokines, through the expression of chemokines (e.g., CCL-2, CCL-20, CXCL-8/IL-8, and CXCL10), cytokines (e.g., IL-1β, TNF-α, IL-6, and IL-17C) [14,15], and surface proteins (e.g., ICAM-1, MHC) [16,17], in order to recruit different classes of leukocytes. In this context, the transcription factor NF-κB plays a fundamental role; in fact, it is involved in the expression of all the aforementioned classes of proinflammatory mediators.

Moreover, keratinocytes may release a relevant number of cytokines in response to the adaptive immune response. For example, different authors have shown that IL-6 production is elevated by IL-4 or IFN-γ in keratinocytes, especially in a histamine-enriched milieu [18,19]; IL-6 may play a dual role in autoimmunity by contributing to the humoral response through the polarization of Th2 and B lymphocytes [20] and promoting Th1/Th17 differentiation [21]. In analogy, Kim et al. [22] conducted excellent work in optimizing a cytokine milieu able to reproduce AD-like inflammation in a model of human keratinocytes (HaCaT). In this cell line, the expression of AD-related biomarkers requires induction with cytokines characteristic of both the innate (TNF-α) and the adaptive (IL-4, IFN-γ) immune responses, thus remarking the complexity of the pathogenesis at the keratinocyte level. In fact, the combination of TNF-α with IFN-γ is required to enhance IL-33 expression, while the combination of TNF-α with IL-4 is necessary to enhance TSLP expression. Notably, both combinations cause a parallel decrease in several indicators of epidermal integrity and differentiation.

As of today, the role of *R. nigrum* leaf extracts against keratinocyte-derived inflammation is still unrevealed. According to recent reviews on the topic, the contribution of flavonols to the biological properties of blackcurrant leaves has only marginally been evaluated by previous authors [23]. The purpose of this work was to investigate the effects of an aqueous extract of *Ribes nigrum* L. leaves in a model of human keratinocytes (HaCaT) challenged with TNF-α, either alone or in combination with the costimulatory cytokines IFN-γ (5 ng/mL) or IL-4 (100 ng/mL). The experimental setting aimed at mimicking Th1 or Th2 type responses, respectively. Following this approach, the preferential activity of the extract against IFN-γ-mediated inflammation was discovered, thus suggesting a potential selectivity against Th1 response.

## 2. Results

### 2.1. Phytochemical Analysis

*Ribes nigrum* leaf extract (RNLE), a dry extract obtained from the aqueous extraction of *Ribes nigrum* L. leaves, was subjected to a phytochemical analysis aimed at identifying the main phenolic compounds, with particular attention paid to flavonoids.

The total phenolic content (TPC) was initially assessed by Folin–Ciocȃlteu assay, which displayed a value (mean ± SD) of 17.260 ± 0.473 mg/g_(extract)_, expressed as gallic acid equivalents.

Quantitative analysis of the extract by LC–MS/MS showed the presence of several flavonoids, especially quercetin and kaempferol derivatives. The percentage of identified phenolic compounds was 13.8%; the occurrence of individual flavonoids/phenolics is reported in Table 1.

### 2.2. RNLE Increases IL-10 Expression in TNF-α-Induced HaCaT Cells

The therapeutic relevance of TNF-α-signaling inhibition in skin inflammation has been highlighted by the clinical efficacy of anti-TNF-α drugs (i.e., etanercept). Consequently, applying a comprehensive approach, the effect of RNLE (100 µg/mL) on TNF-α-challenged HaCaT cells was first evaluated in a multigenic qPCR macroarray.

**Figure 1:**
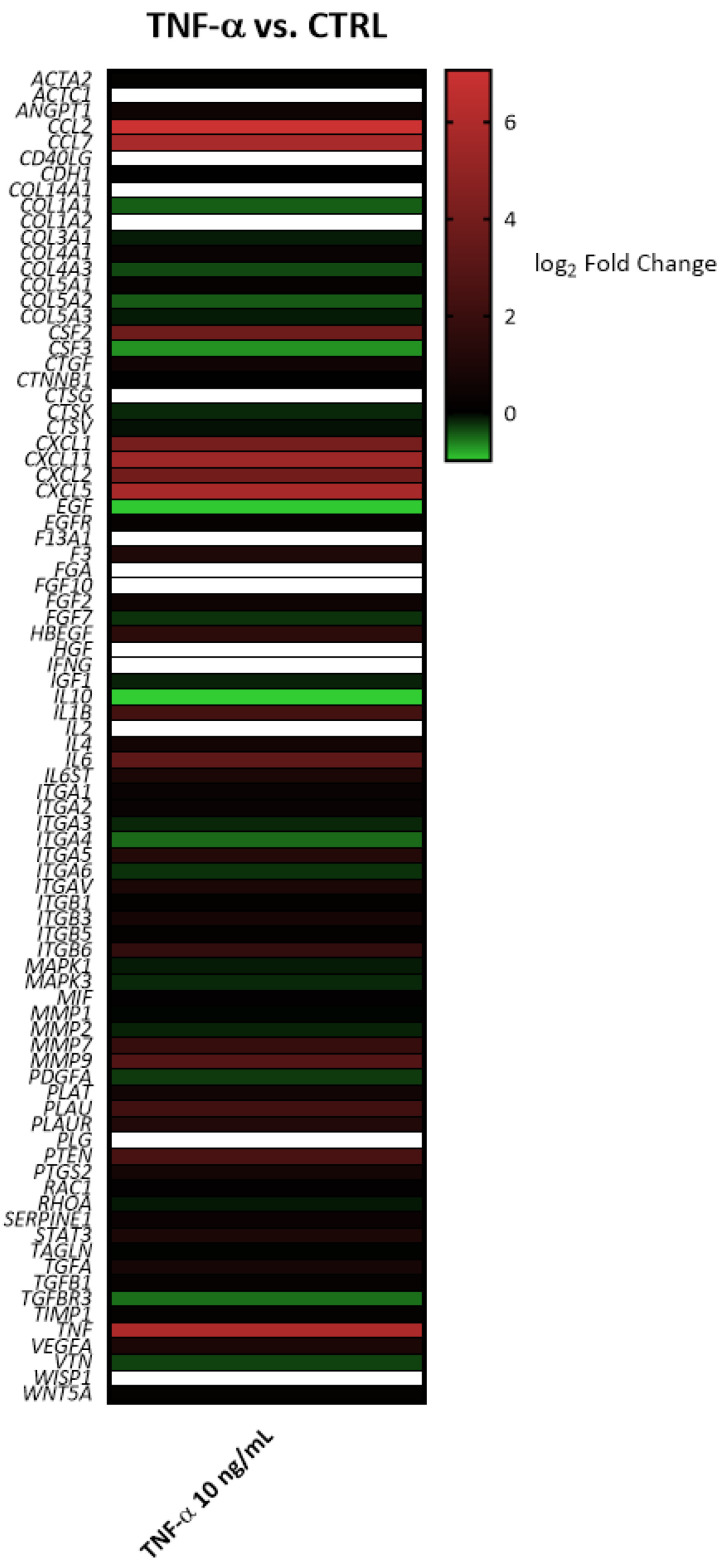
Heatmap depicting the alterations of gene expression induced by TNF-α (10 ng/mL, 6 h). The color scale represents gene upregulation (**red**) and downregulation (**green**) compared to the control (log_2_ fold change = 0, **black**). In white, genes present in the panel but not expressed in the experimental model.

**Figure 2:**
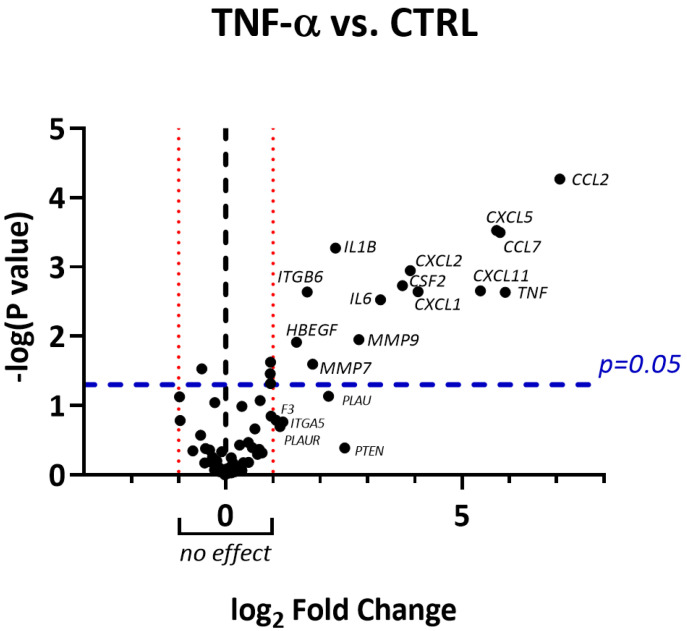
Volcano plot showing the entity of gene up/downregulation induced by TNF-α (10 ng/mL, 6 h) together with statistical significance. Only genes with absolute fold change greater than 2 (log_2_ fold change greater than 1) and *p* < 0.05 were considered significantly regulated.

**Figure 3:**
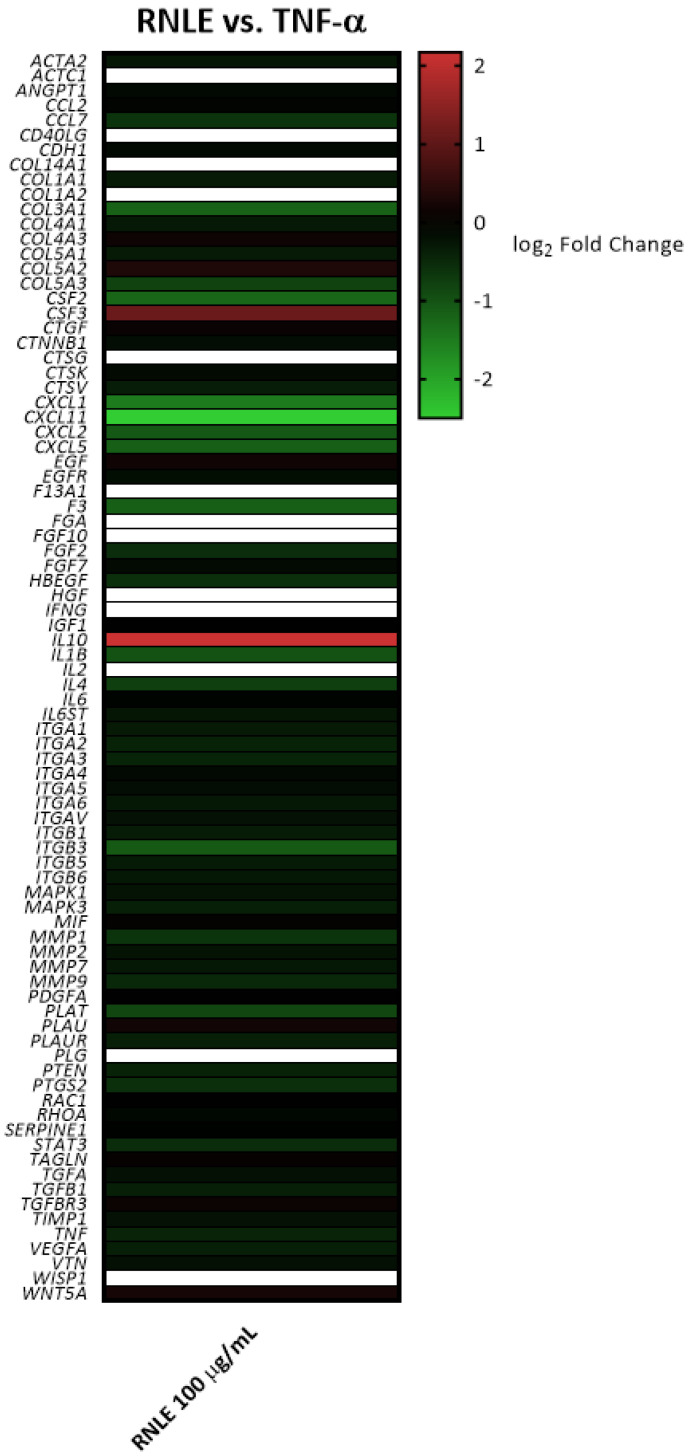
Heatmap depicting the effect on gene expression induced by RNLE treatment (100 µg/mL) in HaCaT cells stimulated with TNF-α (10 ng/mL, 6 h). The color scale represents gene upregulation (**red**) and downregulation (**green**) compared to TNF-α-induced condition (log_2_ fold change = 0, **black**). In white, genes present in the panel but not expressed in the experimental model.

**Figure 4:**
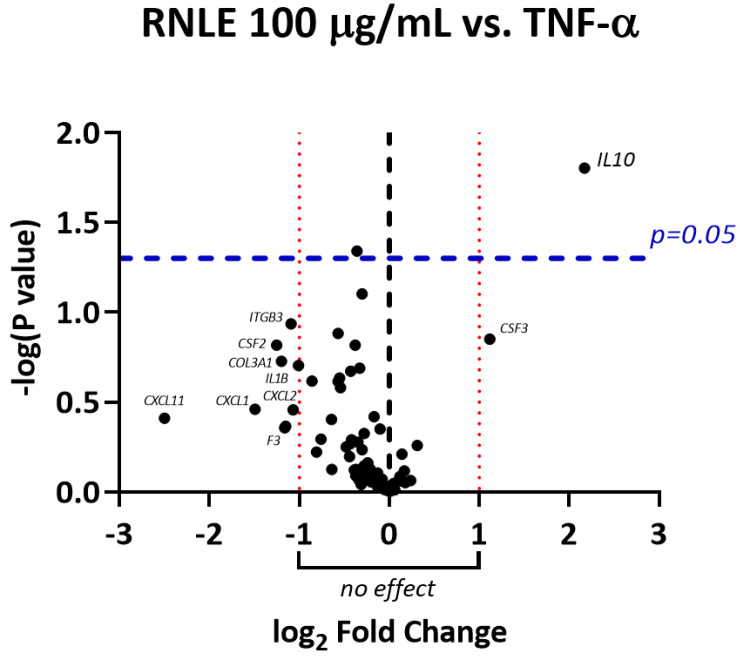
Volcano plot showing the entity of gene up/downregulation induced by RNLE treatment (100 µg/mL) in HaCaT cells stimulated with TNF-α (10 ng/mL, 6 h) together with statistical significance. Only genes with absolute fold change greater than 2 (log_2_ fold change greater than 1) and *p* < 0.05 were considered significantly regulated.

In line with our previous study [14], TNF-α caused the overexpression of genes involved in monocyte and granulocyte recruitment (*CCL2*, *CCL7*, *CSF2*, *CXCL5*, *CXCL1*), as well as in lymphocyte chemoattraction and activation (*CXCL11*, *IL6*) (Figure 1 and Figure 2). RNLE was able to partially revert the upregulation of *CCL7*, *CSF2*, *CXCL1*, and *CXCL11*, although this effect did not reach statistical significance (Figure 2 and Figure 3). It is worth considering that these genes are target of the transcription factor NF-κB [24]. The most peculiar finding was that the strongest inhibitory trend was observed for *CXCL11*, a gene encoding an IFN-γ-inducible chemokine, regulated, similarly to CXCL10 and ICAM-1, by NF-κB and STAT-1 activity [25]. In addition, TNF-α also caused a slight downregulation of the anti-inflammatory *IL10* gene, while the concomitant treatment with RNLE resulted in a significant induction of *IL10* expression (Figure 4), thus suggesting a possible pro-resolving mechanism. However, the overexpression of *IL10* mRNA was not paralleled by an increment in IL-10 protein levels, whether in the intracellular compartments or those secreted in the culture medium (data not shown).

### 2.3. RNLE Inhibits TNF-α/IFN-γ- but Not TNF-α-Induced IL-8 Release in HaCaT Cells

The previous results, regarding gene expression, suggested that RNLE may cover a marginal role against a TNF-α-induced inflammatory profile. However, TNF-α is known to synergize with IFN-γ to enhance a classical Th1 response in keratinocytes, triggering a wider program for pathogen clearance. In particular, IFN-γ is frequently used to prime cells for a stronger response toward TNF-α during in vitro experiments. At a molecular level, the activation of the JAK/STAT-1 pathway by IFN-γ cooperates with the transcriptional activity of NF-κB during TNF-α-induction; thus, the combination of TNF-α and IFN-γ has been shown to strongly induce the expression of IL-8, IL-6, and ICAM-1, considered to be among the most relevant proinflammatory mediators in keratinocytes [26,27].

**Figure 5:**
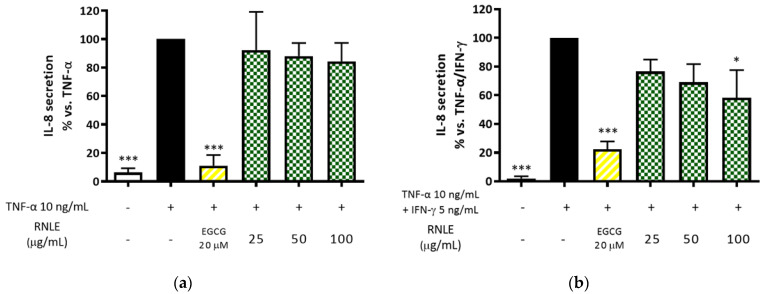
(**a**) Effect of RNLE treatment (25–100 µg/mL) on IL-8 secretion in HaCaT cells stimulated with TNF-α (10 ng/mL, 24 h); the amount of IL-8 in the stimulated condition was 211.31 ± 34.26 pg/mL. (**b**) Effect of RNLE treatment (25–100 µg/mL) on IL-8 secretion in HaCaT cells stimulated with a combination of IFN-γ and TNF-α (5 ng/mL + 10 ng/mL, 24 h); the amount of IL-8 in the stimulated condition was 1084.66 ± 118.62 pg/mL. (−)-Epigallocatechin 3-gallate (EGCG) 20 µM was used as the reference compound; * *p* < 0.05, *** *p* < 0.001 vs. stimulus.

As a consequence, we performed a series of experiments aimed at assessing the effect of RNLE (25–100 µg/mL) on the synergic contribution of IFN-γ in TNF-α-challenged HaCaT cells. RNLE inhibited IL-8 release following stimulation with a combination of both cytokines, but not when TNF-α was used alone (Figure 5).

In line with the inhibitory trend observed for *CXCL11* expression, this experiment suggests that RNLE may preferentially interfere with IFN-γ, rather than TNF-α, signaling.

### 2.4. RNLE Inhibits TNF-α/IFN-γ-Induced IL-6 Release in HaCaT Cells

Accordingly, we evaluated the effect of RNLE on the release of IL-6 in TNFα/IFN-γ-challenged HaCaT cells. Notably, preliminary time-course experiments (Figure A3, Appendix A) showed that IFN-γ (5 ng/mL) was necessary for a considerable induction of IL-6 release (149.52 ± 35.48 pg/mL) by TNF-α (10 ng/mL), which could not be achieved (35.67 ± 9.73 pg/mL) by TNF-α alone. On the other hand, IFN-γ alone was not sufficient to induce the release of the cytokine, thus reflecting its priming role in the response to TNF-α in this experimental setting. In this sense, the regulation of IL-6 release displays a marked difference if compared to IL-8, for which TNF-α by itself is sufficient to obtain a considerable induction.

**Figure 6:**
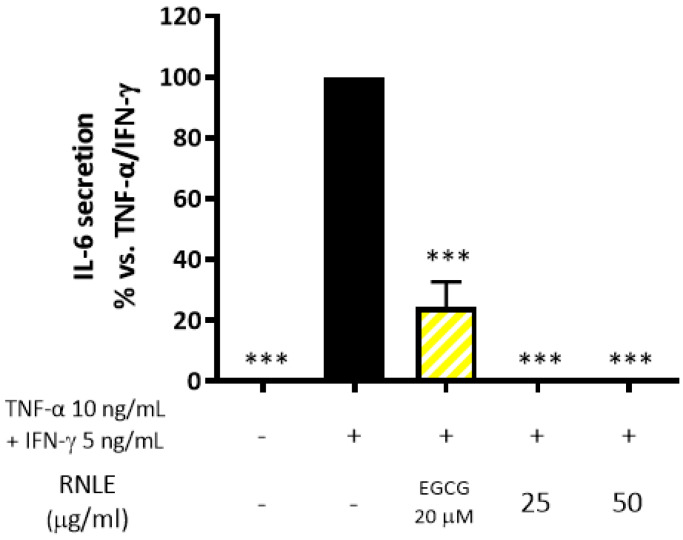
Effect of RNLE treatment (25–50 µg/mL) on IL-6 secretion in HaCaT cells stimulated with a combination of IFN-γ and TNF-α (5 ng/mL + 10 ng/mL, 24 h); the amount of IL-6 in the stimulated condition was 118.66 ± 15.95 pg/mL. (−)-Epigallocatechin 3-gallate (EGCG) 20 µM was used as the reference compound; *** *p* < 0.001 vs. stimulus.

RNLE (25–50 µg/mL) totally abrogated IL-6 release (Figure 6). In line with the experimental setting, the suppression of IFN-γ signaling would have accounted for a stronger inhibitory effect on IL-6 rather than IL-8 release. Consequently, these results reinforced the hypothesis of a preferential activity of RNLE against IFN-γ signaling.

### 2.5. RNLE Preferentially Inhibits IFN-γ- over TNF-α-Induced s-ICAM-1 Release in HaCaT Cells

ICAM-1 is a cell-surface adhesion molecule which allows the interaction between cells and leukocytes at the site of inflammation. Its soluble form (s-ICAM-1) is produced after cleavage and represents a well-established marker of cellular activation, strongly correlated with the induction of ICAM-1 expression [28]. As previously demonstrated in the scientific literature, ICAM-1 is induced by TNF-α and IFN-γ in many cellular types including keratinocytes [16,29]. Notably, IFN-γ was reported to elicit ICAM-1 expression at lower concentration than TNF-α in HaCaT cells [17]. In our experiments, we could observe an analogous behavior, since a concentration of IFN-γ half that of TNF-α led to a similar quantitative release of s-ICAM-1 (422.97 ± 90.28 and 512.99 ± 84.17 pg/mL for IFN-γ and TNF-α, respectively).

**Figure 7:**
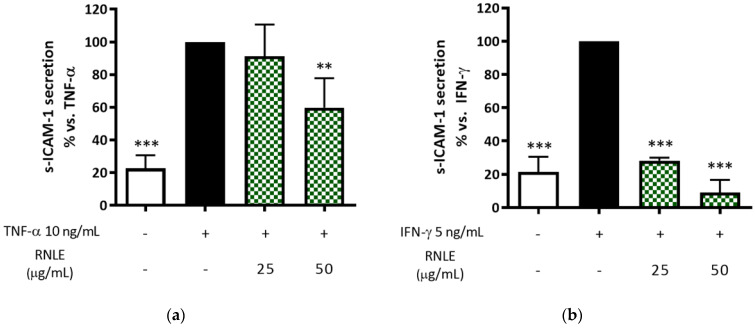
(**a**) Effect of RNLE treatment (25–50 µg/mL) on s-ICAM-1 secretion in HaCaT cells stimulated with TNF-α (10 ng/mL, 24 h); the amount of s-ICAM in the stimulated condition was 512.99 ± 84.17 pg/mL. (**b**) Effect of RNLE treatment (25–50 µg/mL) on s-ICAM-1 secretion in HaCaT cells stimulated with IFN-γ (5 ng/mL, 24 h); the amount of s-ICAM in the stimulated condition was 422.97 ± 90.28. ** *p* < 0.01, *** *p* < 0.001 vs. stimulus.

RNLE (25–50 µg/mL) inhibited both TNF-α- and IFN-γ-induced s-ICAM-1 release. Nevertheless, this effect was displayed in a concentration-dependent fashion with a preferential impairment of IFN-γ versus TNF-α challenge (Figure 7), thus confirming our previous observations. The vascular protective activity of the flavonols present in RNLE, including the inhibition of adhesion molecules, is well known [30,31]. To the best of our knowledge, the bioactivity of flavonols against ICAM-1 expression has been less investigated in keratinocytes than in other cellular populations. Bito et al. showed that quercetin and taxifolin counteract IFN-γ-induced ICAM-1 expression in keratinocytes, thus suggesting the potential involvement of flavonols in RNLE activity [32].

### 2.6. RNLE Inhibits TNF-α/IFN-γ-Induced NF-κB Activity in HaCaT Cells

As previously mentioned, NF-κB plays a pivotal role in the promoter activity of IL-8, IL-6, and ICAM-1. Consequently, in the subsequent experiments, we wondered whether an interference in this mechanism might account for the activity of RNLE. Following the previous approach, HaCaT cells were treated with RNLE concomitantly with TNF-α or TNF-α/IFN-γ stimulation.

**Figure 8:**
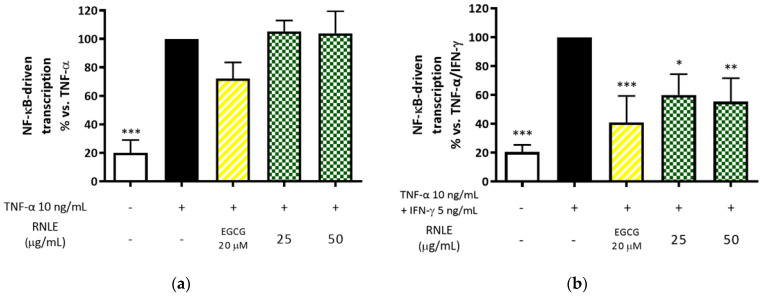
(**a**) Effect of RNLE treatment (25–50 µg/mL) on NF-κB-driven transcription in HaCaT cells stimulated with TNF-α (10 ng/mL, 24 h). (**b**) Effect of RNLE treatment (25–100 µg/mL) on NF-κB-driven transcription in HaCaT cells stimulated with a combination of IFN-γ and TNF-α (5 ng/mL + 10 ng/mL, 24 h). (−)-Epigallocatechin 3-gallate (EGCG) 20 µM was used as the reference compound; * *p* < 0.05, ** *p* < 0.01, *** *p* < 0.001 vs. stimulus.

In line with the aforementioned inhibition of IFN-γ-induced inflammatory markers, the activity of NF-κB was impaired by a concentration of RNLE as low as 25 µg/mL under TNF-α/IFN-γ stimulation; on the contrary, the extract was unable to counteract NF-κB activity under TNF-α stimulation (Figure 8). This evidence demonstrates that RNLE may counteract IFN-γ signaling at least at a transcriptional level, thus arousing interest in the use of blackcurrant leaves against Th1 inflammation in skin.

### 2.7. RNLE Is Not Able to Inhibit TNF-α/IL-4-Induced IL-6 Release in Differentiated HaCaT Cells

At this point of the work, we decided to shift our focus to the initial question regarding the possible selectivity of RNLE against Th1 or Th2 type responses in keratinocytes. At first, we observed that RNLE was able to enhance mRNA but not protein levels of IL-10, a widely recognized pro-resolving type 2 mediator. Then, after having found that IFN-γ-inducible mediators were conversely impaired by the extract, we moved to the investigation of the bioactivity against TNF-α/IL-4 stimulation as a model of Th2 inflammation.

In the last few years, the scientific research has elucidated the ways in which inflammation and differentiation are interweaved in keratinocytes. According to the literature, IL-6 not only plays a critical role in the differentiation of the lymphocyte sub-population involved in skin autoimmunity [10], but also acts on keratinocytes by impairing their differentiation [33]. In fact, IL-6 and IL-4 were shown to downregulate markers of early (keratin-1, keratin-10) and terminal (involucrin) differentiation [34,35]. Consequently, we selected IL-6 release as a suitable outcome for either the investigation of RNLE activity against TNF-α/IL-4 stimulation and the dissection of the role of RNLE in Th1 or Th2 response.

For this purpose, we differentiated HaCaT cells by maintaining HaCaT cells under validated cultural conditions [34,36] and then verified the effect of TNF-α/IL-4 stimulation on IL-6 release during the differentiation time (from 3 to 11 days). IL-4 enhanced the effect of TNF-α on IL-6 release, and their synergy was magnified with the progression of differentiation. Notably, in this context, TNF-α alone was sufficient to induce IL-6 release, but secreted levels (224.60 ± 47.02 pg/mL) did not increase over the differentiation period (Figure A4, Appendix A). Although the involvement of IL-4 in the alteration of the epidermal barrier has been elucidated, studies regarding the role of differentiation in the response to IL-4 in keratinocytes were not extensive. We did not further investigate the molecular machinery behind the contribution of the differentiation to the effect of IL-4; however, Nishio et al. [37] demonstrated that proteins downstream of IL-4 receptor (JAK2/STAT-6) are more expressed in the granular layer of the healthy skin than in the lower layers. Differentiated HaCaT cells are referred to as a model of early differentiation; thus, theoretically, the increase in JAK2/STAT-6 expression during differentiation is plausible and may enhance the IL-4 response.

**Figure 9:**
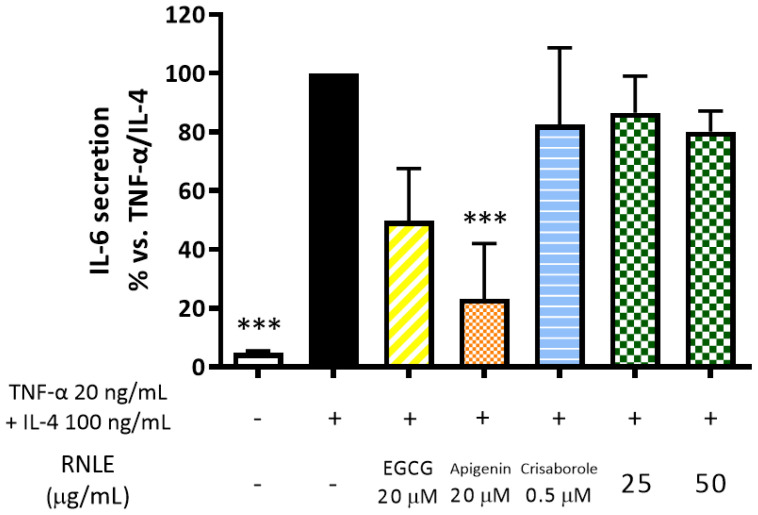
Effect of RNLE treatment (25–50 µg/mL) on IL-6 secretion in differentiated HaCaT cells stimulated with a combination of IL-4 and TNF-α (100 ng/mL + 20 ng/mL, 24 h); the absolute amount of IL-6 in the stimulated condition was 612.59 ± 59.55 pg/mL. (−)-Epigallocatechin 3-gallate (EGCG) 20 µM, apigenin 20 µM, and crisaborole 0.5 µM were used as the reference compounds; *** *p* < 0.001 vs. stimulus.

Consequently, we evaluated the effect of RNLE on differentiated cells, stimulated by the combination of TNF-α and IL-4. In contrast to previous observations, RNLE (25–50 µg/mL) was unable to significantly impair IL-6 release in this experimental setting (Figure 9). The result suggested a marginal impact of RNLE on IL-4 signaling.

### 2.8. RNLE Inhibits TNF-α/IL-4-Induced TSLP Release in Differentiated HaCaT Cells with a cAMP-Independent Mechanism

To more deeply evaluate the actual role of RNLE in Th2 response, we selected the cytokine TSLP, i.e., another type 2 cytokine involved in atopic inflammation through the activation of dendritic and ILC2 cells, as an additional outcome [13]. In AD and psoriatic patients, TSLP has been shown to preferentially accumulate in the upper layers of the epidermis and, among skin cells, originates exclusively from keratinocytes [22,38]. Moreover, in analogy with IL-6, its levels correlate with the impairment of epidermal integrity [39].

According to our experiments, TSLP was released (65.05 ± 31.91 pg/mL) by HaCaT cells only late in the differentiation period and required the combination of TNF-α and IL-4. This result was concordant with the experiments conducted on inflamed human skin explants by Bogiatzi et al. [38], in terms of the amount of TSLP release and the prerequisite of differentiation for this effect to be observed.

**Figure 10:**
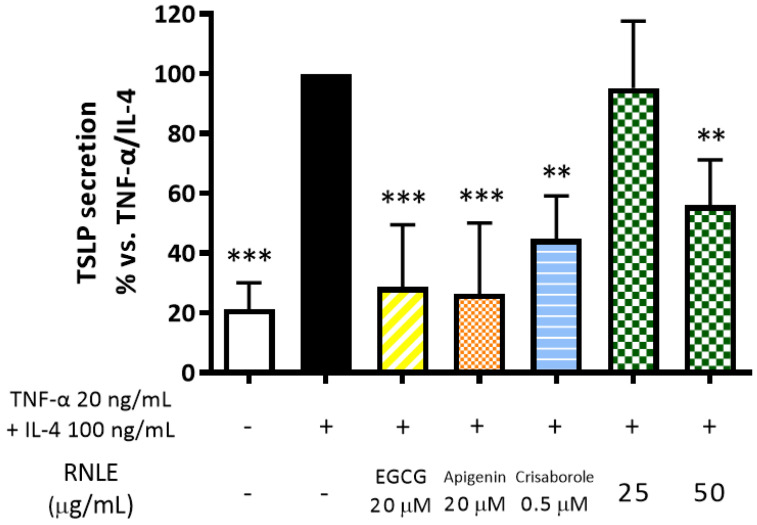
Effect of RNLE treatment (25–50 µg/mL) on TSLP secretion in differentiated HaCaT cells stimulated with a combination of IL-4 and TNF-α (100 ng/mL + 20 ng/mL, 24 h); the absolute amount of TSLP in the stimulated condition was 65.05 ± 31.91 pg/mL. (−)-Epigallocatechin 3-gallate (EGCG) 20 µM, apigenin 20 µM, and crisaborole 0.5 µM were used as the reference compounds; ** *p* < 0.01, *** *p* < 0.001 vs. stimulus.

Consequently, the effect of RNLE on TSLP was evaluated in differentiated HaCaT cells stimulated with the combination TNF-α/IL-4, under the same experimental setting applied for the investigation of IL-6 release. In these conditions, RNLE inhibited TSLP release by 56% at a concentration of 50 µg/mL (Figure 10).

The molecular machinery responsible for TSLP release by keratinocytes is not completely clear, but NF-κB and NFAT may play an important role in TSLP transcription in response to mechanical and biological injuries [40,41].

The pharmacological inhibition of PDE4 and the elevation of intracellular cAMP levels were demonstrated to impair the proinflammatory activity of transcription factors (such as NFAT and NF-κB), leading to the inhibition of cytokine production, including TSLP [42,43]. Moreover, cAMP elevation was associated with keratinocyte differentiation [44]. Notably, many flavonoids are well-known NF-κB inhibitors and also possess inhibitory activity on different PDE isoforms, including PDE4 [45,46]. Among them, the flavone apigenin was shown, on the one hand, to non-selectively inhibit PDE4 [47] and, on the other, to reduce the levels of proallergic cytokines in models of asthma and atopic dermatitis [48,49]; however, a causal link between the two effects could not be established. For these reasons, we decided to further correlate the effect of RNLE with that of crisaborole (0.5 µM), a topical PDE4 inhibitor approved in 2016 by FDA for the treatment of AD. Apigenin (20 µM) was included in the experiments as a representative flavonoid.

**Figure 11:**
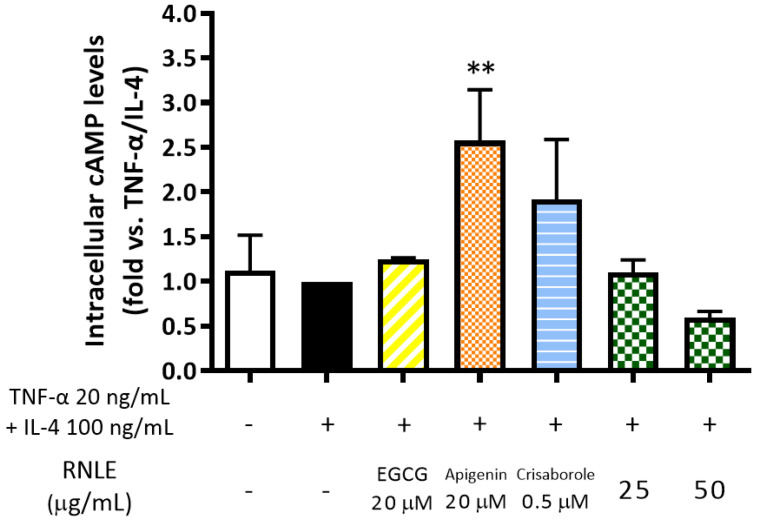
Effect of RNLE treatment (25–50 µg/mL) on intracellular cAMP levels in differentiated HaCaT cells stimulated with a combination of IL-4 and TNF-α (100 ng/mL + 20 ng/mL, 24 h). (−)-Epigallocatechin 3-gallate (EGCG) 20 µM, apigenin 20 µM, and crisaborole 0.5 µM were used as the reference compounds; ** *p* < 0.01 vs. stimulus.

Since crisaborole and apigenin exhibited a strong inhibitory effect on TSLP release (Figure 8), we speculated that the bioactivity of RNLE might involve a cAMP-dependent mechanism. Consequently, we measured intracellular cAMP levels in the same experimental setting. Apigenin and crisaborole, as expected, caused an increase in intracellular cAMP levels, whereas RNLE could not induce this molecular mechanism (Figure 11). However, EGCG (20 µM), used as a positive control in each experiment, showed the ability to impair TSLP release in the absence of cAMP elevation, thus suggesting that RNLE may act through cAMP-independent mechanisms as well.

### 2.9. RNLE Slightly Inhibits TNF-α/IL-4-Induced NF-κB Activity in HaCaT Cells

**Figure 12:**
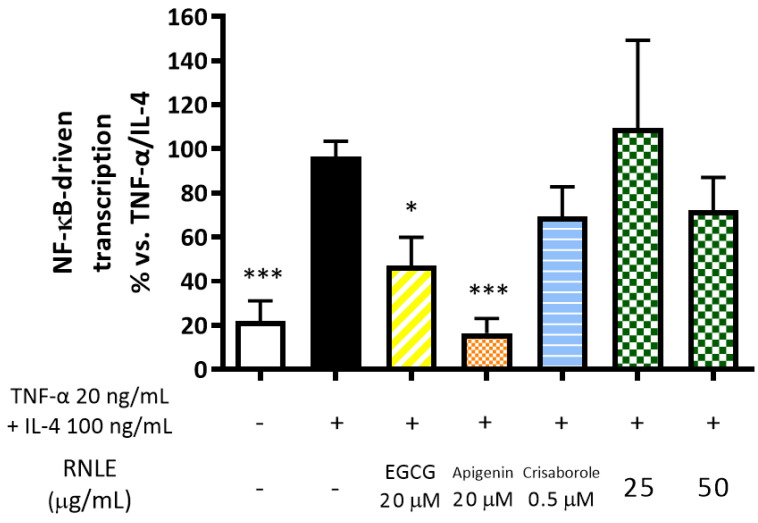
Effect of RNLE treatment (25–50 µg/mL) on NF-κB-driven transcription in HaCaT cells stimulated with a combination of IL-4 and TNF-α (100 ng/mL + 20 ng/mL, 6 h). (−)-Epigallocatechin 3-gallate (EGCG) 20 µM, apigenin 20 µM, and crisaborole 0.5 µM were used as the reference compounds; * *p* < 0.05, *** *p* < 0.001 vs. stimulus.

NF-κB has been demonstrated to regulate both TSLP and IL-6 expression. However, TSLP and IL-6 release was differently modulated by RNLE treatment in our experiments; thus, we decided to investigate the effect of the extract on NF-κB activation to better clarify its involvement. In line with the substantial absence of an inhibitory effect on IL-6 release and the lack of cAMP-elevating activity, RNLE was not able to counteract TNF-α/IL-4-induced NF-κB-driven transcription, although a slight but not statistically significant inhibition was observed at 50 µg/mL (Figure 12).

It is intriguing to note that RNLE at a concentration of 50 µg/mL paralleled the effect of crisaborole (0.5 µM) with regard to all TNF-α/IL-4-related parameters (IL-6, TSLP, NF-κB-driven transcription), with the exception of cAMP levels. This observation suggests that the effect of RNLE on TSLP release might not be due directly to cAMP levels, but rather to the cAMP-independent activity on downstream cAMP-dependent proteins and cAMP sensors with anti-inflammatory implications, such as PKA and EPAC (see the reviews by Murray [50] and Woo and Kuzel [51]). In line with this hypothesis, several authors have demonstrated that flavonols may activate PKA- and EPAC-dependent pathways [52,53]. Notably, the activation of PKA/CREB signaling may also contribute to the overexpression of *IL-10* [54], as previously observed, thus reinforcing the hypothesis that the activity of the extract under study might be potentially due to flavonols.

### 2.10. RNLE Is Not Able to Inhibit Histamine-Induced IL-6 Release in HaCaT Cells

TSLP involved is involved in not only Th2 cell maturation but also itch elicitation. Its action is either direct on sensory neurons [55] or indirect through dendritic, ILC2, and mast-cell stimulation [13]. Histamine is a key mediator of itch and allergic inflammation, which has been shown to enhance the release of several cytokines by inflamed keratinocytes, including TSLP and IL-6 [18,19].

**Figure 13:**
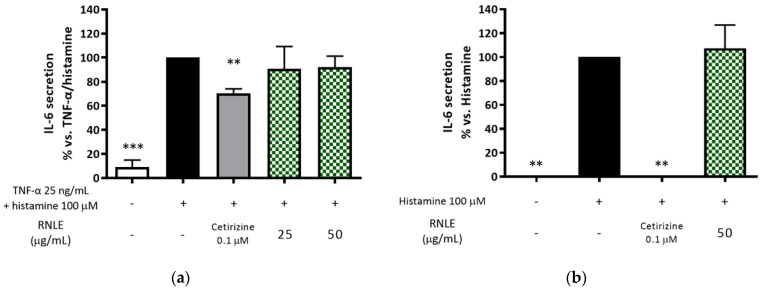
(**a**) Effect of RNLE treatment (25–50 µg/mL) on IL-6 secretion in HaCaT cells stimulated with a combination of histamine and TNF-α (100 µM + 25 ng/mL, 24 h); the amount of IL-6 in the stimulated condition was 64.50 ± 25.85 pg/mL. (**b**) Effect of RNLE treatment (25–50 µg/mL) on IL-6 secretion in HaCaT cells stimulated with histamine (100 µM, 24 h); the amount of IL-6 in the stimulated condition was 3.20 ± 1.10 pg/mL. Cetirizine 0.1 µM was used as the reference compound; ** *p* < 0.01, *** *p* < 0.001 vs. stimulus.

In line with the findings of Wölfle et al. [56], we verified that IL-6 release is effectively induced by the combination of histamine (100 µM) and TNF-α in HaCaT cells. Consequently, we assessed if RNLE might also counteract the signaling of histamine, by selecting IL-6 as the experimental outcome. As shown in Figure 13, RNLE was unable to inhibit histamine alone or histamine/TNF-α-stimulated IL-6 secretion in comparison with cetirizine (0.1 µM).

These results suggest that RNLE might not counteract itching by inhibiting histamine signaling in keratinocytes. Nevertheless, several authors have reported that, in a wider scenario, flavonols and their glycosides may impact histamine release and humoral responses [57,58], thus proposing a potential antiallergic activity toward other cellular populations.

### 2.11. Quercetin and Kaempferol Preferentially Inhibit IFN-γ-Induced s-ICAM-1 Release in HaCaT Cells

At this point of our work, we wondered if the bioactivity of RNLE could be attributed to the main flavonoids detected in the extract. For this purpose, we selected quercetin and kaempferol (0.1–1 µM) as representative pharmacophores of the parent glycosidic flavonols. Then, we treated HaCaT cells with the flavonols in the presence of TNF-α or IFN-γ to observe the effect on s-ICAM-1 release, thus allowing a reliable comparison with the experiments showed in Figure 7.

**Figure 14:**
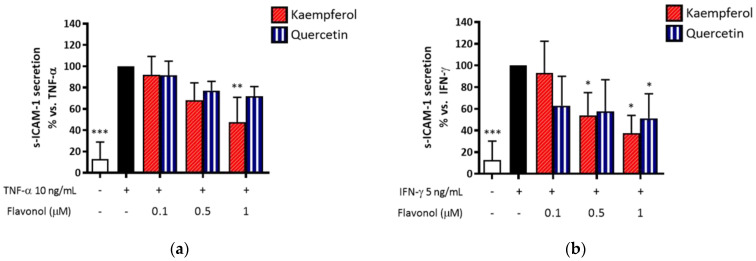
(**a**) Effect of quercetin and kaempferol treatment (0.1–1 µM) on s-ICAM-1 release in HaCaT cells stimulated with TNF-α (10 ng/mL, 24 h). (**b**) Effect of quercetin and kaempferol treatment (0.1–1 µM) on s-ICAM-1 release in HaCaT cells stimulated with IFN-γ (5 ng/mL, 24 h). * *p* < 0.05, ** *p* < 0.01, *** *p* < 0.001 vs. stimulus.

Quercetin and kaempferol preferentially inhibited IFN-γ-induced s-ICAM-1 release in a concentration-dependent fashion, thus resembling the activity of RNLE (Figure 14). Furthermore, their inhibitory concentration range was representative of that achievable by RNLE treatment, as evident from the quantitative analysis. However, kaempferol resulted more potent than quercetin against both stimulations with either TNF-α (IC_50_ = 0.77 and IC_50_ > 1 µM, respectively) or IFN-γ (IC_50_ = 0.46 and IC_50_ > 1 µM, respectively).

## 3. Conclusions

In the present work, a flavonol-rich blackcurrant leaf extract (RNLE) was discovered to preferentially counteract IFN-γ-mediated, rather than IL-4 mediated, inflammation in keratinocytes. RNLE strongly inhibited the release of IL-6 following TNF-α/IFN-γ, but not TNF-α/IL-4 challenge. The effect against IFN-γ challenge was also highlighted by the strong inhibition of IFN-γ-mediated s-ICAM-1 release, recognized as a marker of cell-mediated immunity in skin. This effect was attributed to the aglycones of the main flavonoids present in the extract: quercetin and kaempferol. Nevertheless, the extract was also able to inhibit TNF-α/IL-4-induced TSLP release, thus opening questions related to the potential impact on Th1/Th2 balance in inflammatory-based skin diseases.

Although the inhibition of NF-κB activity explained, at least in part, the mechanism of action, our work demands further dermatological studies involving other potential molecular targets for flavonols, such as JAK/STAT and PKA signaling. The inhibition of IL-6 and TSLP release suggests an alternative antiallergic and antipruritic effect, which can add to the known antihistaminic effect of flavonols on skin cell populations other than keratinocytes. The findings of our study ascribe to *R. nigrum* leaf extract anti-inflammatory activities that could be exploited and may exert synergic effects in association with other blackcurrant products, such as seed oil, widely recognized for its promising beneficial effects at a cutaneous level due to the presence of γ-linolenic acid.

A deeper molecular investigation may clarify the role of blackcurrant leaf extracts in specific inflammatory disturbances, in which IFN-γ plays a central role, such as psoriasis and arthritis, one of the traditional indications reported in the ESCOP monograph.

## 4. Materials and Methods

*Ribes nigrum* leaf extract (RNLE) is a dry extract obtained from the extraction of *Ribes nigrum* L. leaves with aqueous solvent (extract to drug ratio 1:3). The extract was kindly provided by Drex Pharma S.r.l., via Privata Tarvisio, 32–20,125 Milano (MI) Italy.

### 4.1. Cell Culture

HaCaT (CVCL-0038; Cell Line Service, Eppelheim, Germany), a spontaneously immortalized human keratinocyte cell line from adult skin [59], was maintained at 37 °C, in a humidified atmosphere containing 5% CO_2_, in Dulbecco’s modified Eagle’s medium (DMEM; Merck Life Science, Milano, Italy) supplemented with 10% heat-inactivated fetal bovine serum (Euroclone, Pero, Italy), 100 U/mL penicillin, 100 µg/mL streptomycin (Pen Strep Gibco^TM^; Thermo Fisher Scientific, Monza, Italy), and 2 mM l-glutamine (Thermo Fisher Scientific, Monza, Italy). Every 4 days, cells were detached from 75 cm^2^ flasks (Primo^®^; Euroclone, Pero, Italy) using Trypsin-EDTA 0.25% (Gibco^TM^; Thermo Fisher Scientific, Monza, Italy), counted, and sub-cultured in a new flask or seeded in 24-well plates (Falcon^®^; Corning Life Sciences, Amsterdam, Netherlands) for the biological tests.

### 4.2. Cell Treatments

HaCaT were seeded at a density of 6 × 10^4^ cells/well in 24-well flat-bottom multiwell plates (Falcon^®^; Corning Life Sciences, Amsterdam, Netherlands). After 72 h of growth, cells were treated with RNLE, alone, to assess cytotoxicity, or in the presence of different proinflammatory stimuli, i.e., human recombinant TNF-α (Peprotech, London, UK), alone or in combination with IFN-γ (PeproTech, London, UK), IL-4 (Peprotech, London, UK), or histamine (Merck Life Science, Milano, Italy). For the assessment of specific parameters, HaCaT cells were differentiated by maintaining HaCaT cells in multiwell plates under the previously stated cultural conditions over a period of 11 days for time course experiments [34,36]. In particular, differentiated cells were grown under high-calcium medium (DMEM, supplemented as stated in the previous section with the with the addition of Ca^2+^ 1.8 mM) for 9 days, replacing the medium every 48 h, and then treated for 24 h as mentioned below. The differentiation paradigm was previously validated by the assessment of cytokeratin-10 and involucrin protein synthesis (data not shown).

Depending on the biological parameter to be evaluated, the following specific treatment protocols were used:

- TNF-α 10 ng/mL, 6 h: assessment of gene expression; IL-8, IL-10, and s-ICAM-1 release; NF-κB-driven transcription.
- TNF-α 10 ng/mL, 24 h: assessment of s-ICAM-1 release.
- TNF-α 10 ng/mL + IFN-γ 5 ng/mL or IFN-γ 5 ng/mL, 24 h: assessment of IL-8, IL-6, and s-ICAM-1 release; NF-κB-driven transcription.
- TNF-α 25 ng/mL + histamine 100 µM or histamine 100 µM alone, 24 h: assessment of IL-6 release.
- TNF-α 20 ng/mL + IL-4 100 ng/mL, 24 h, differentiated cells: assessment of IL-6 and TSLP release; intracellular cAMP levels.
- TNF-α 20 ng/mL + IL-4 100 ng/mL, 6 h: assessment of NF-κB-driven transcription.

Crisaborole (Merck Life Science, Milano, Italy) 0.5 μM, cetirizine (Merck Life Science, Milano, Italy) 0.1 μM, (−)-epigallocatechin 3-gallate (EGCG) (PhytoLab, Vestenbergsgreuth, Germany) 20 μM, and apigenin (Sequoia Research Product, Pangbourne, UK) 20 μM were used as reference inhibitory compounds. EGCG was used in all the experiments involving NF-κB activation and the release of proinflammatory mediators [14], whereas crisaborole/apigenin and cetirizine were specifically used in experiments assessing cAMP and histamine contribution, respectively.

All treatments were performed using Dulbecco’s modified Eagle’s medium (DMEM; Merck Life Science, Milano, Italy) supplemented with 100 U/mL penicillin, 100 µg/mL streptomycin (Pen Strep Gibco^TM^; Thermo Fisher Scientific, Monza, Italy) and 2 mM l-glutamine (Thermo Fisher Scientific, Monza, Italy). At the end of the treatments, media or cell lysates were collected and stored at −20 °C until the subsequent biological assays.

### 4.3. Cytotoxicity and Viability Assays

The integrity of cellular morphology, before and after the treatment, was assessed by light microscopy.

#### 4.3.1. Neutral Red Uptake (NRU) Assay

Cell viability was evaluated using the neutral red (3-amino-7-dimethylamino-2-methyl-phenazine hydrochloride; Merck Life Science, Milano, Italy) uptake assay, adapted from Repetto et al. [60]. After 24 h of incubation in presence of increasing concentrations of RNLE, the culture medium was substituted with 200 µL of neutral red medium (40 µg/mL), and cells were further incubated for 2 h at 37 °C. Then, medium was discarded, cells were thoroughly washed with PBS, and 200 µL of destaining solution (EtOH 50% *v/v* + 1% *v/v* glacial acetic acid) was added to each well. The plate was kept on a plate shaker for 10 min, 100 µL of medium was taken from each well and transferred to a 96-well plate, and the absorbance was measured with an EnVision Multimode Plate Reader (Perkin Elmer, Milano, Italy) at a wavelength of 535 nm.

#### 4.3.2. LDH Assay

The potential cytotoxic effect of RNLE was determined in terms of release of lactate dehydrogenase (LDH) from cells into the extracellular medium using the LDH Cytotoxicity Detection Kit (Takara Bio Europe, Saint-Germain-en-Laye, France), following the manufacturer’s instructions. To overcome the limitations due to the underestimation of the proportion of dead cells in conditions with growth inhibition, a modified protocol including additional condition-specific controls, consisting of the measurement of maximal LDH release for each individual treatment, was used [61]. After 24 h of incubation in presence of increasing concentrations of RNLE, Triton X-100 was added to condition-specific control wells (final concentration 1%) in order to obtain maximal LDH release, and the plate was centrifuged at 250× *g* for 10 min. Then, 50 µL of supernatant was taken from each well, transferred to a 96-well plate, diluted with 50 µL of water, and mixed with 100 µL of reaction mixture. After 30 min of incubation at room temperature protected from light, the absorbance of the sample was measured with a VICTOR X3 Multilabel Plate Reader (Perkin Elmer, Milano, Italy) at a wavelength of 490 nm. Percentage cytotoxicity was calculated according to the equation proposed by Smith et al. [61].

**Figure A1:**
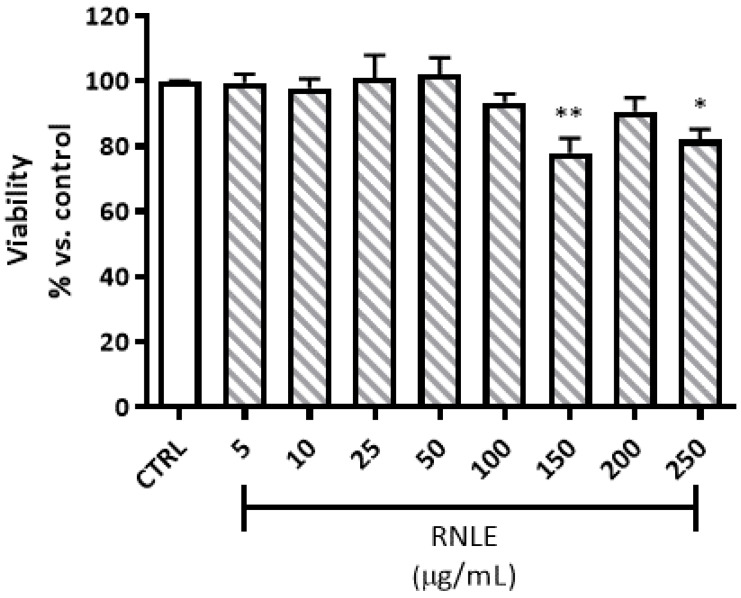
Effect of 24 h RNLE treatment (5–250 µg/mL) on HaCaT cell viability, as measured with Neutral Red Uptake (NRU) assay; * *p* < 0.05, ** *p* < 0.01 vs. stimulus.

**Figure A2:**
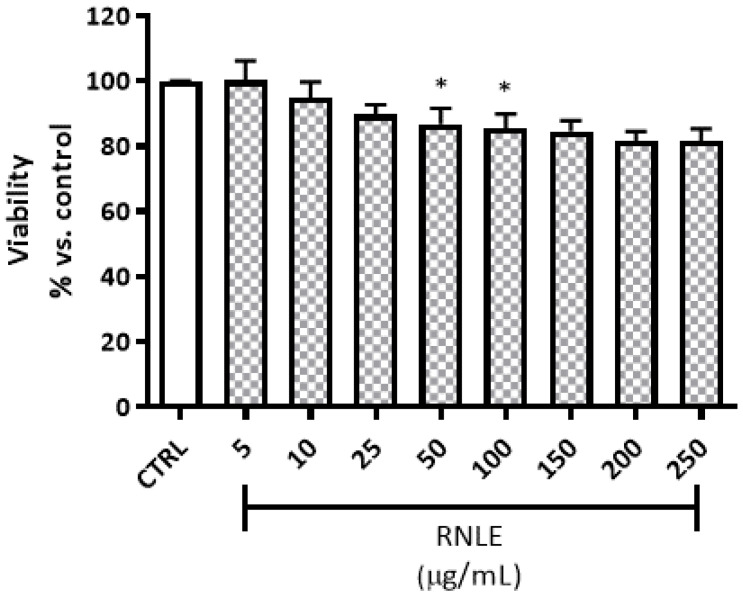
Effect of 24 h RNLE treatment (5–250 µg/mL) on HaCaT cell viability, as measured with LDH assay; * *p* < 0.05 vs. stimulus.

**Figure A3:**
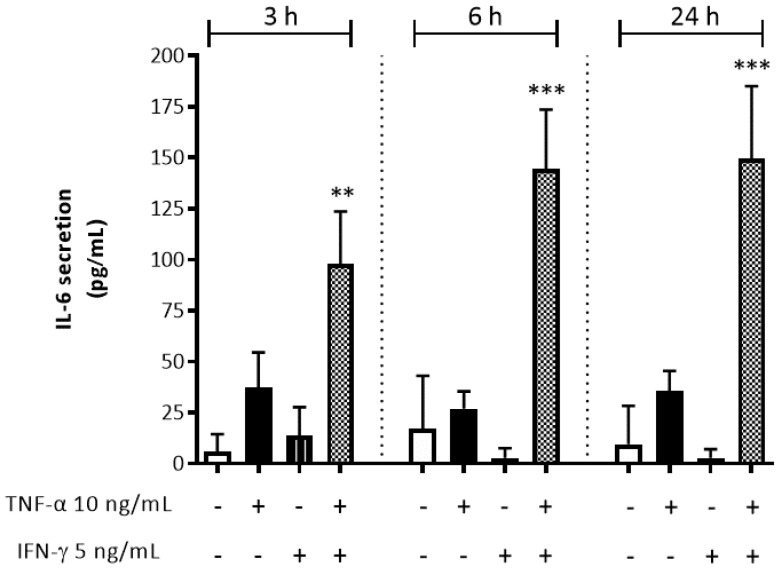
Preliminary time course experiment aimed at assessing IL-6 release from HaCaT cells stimulated with TNF-α (10 ng/mL), IFN-γ (5 ng/mL), or a combination of both; ** *p* < 0.01, *** *p* < 0.001 vs. stimulus.

**Figure A4:**
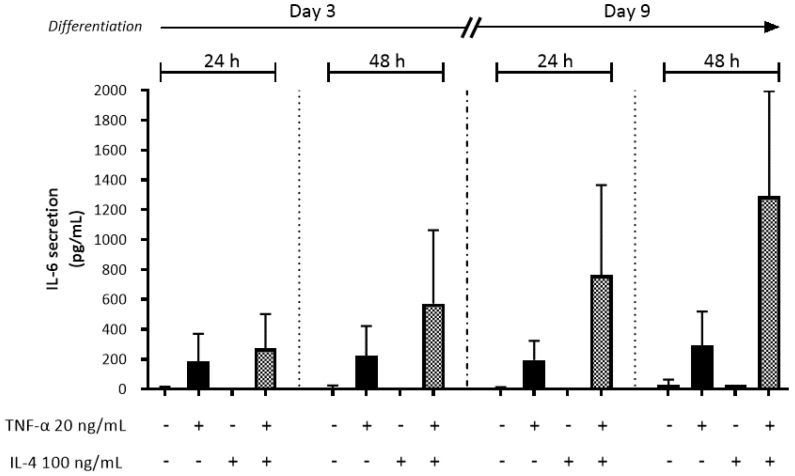
Preliminary time course experiment aimed at assessing IL-6 release from HaCaT cells at different stages of differentiation, stimulated with TNF-α (20 ng/mL), IL-4 (100 ng/mL), or a combination of both.

The extract did not show any cytotoxic effect in the NRU assay in the range of tested concentrations (5–250 μg/mL). Absence of cytotoxicity was confirmed by lactate dehydrogenase (LDH) assay (Figure A1 and Figure A2, Appendix A).

### 4.4. Phytochemical Characterization

#### 4.4.1. Folin–Ciocȃlteu Assay

To assess the total phenolic content (TPC), RNLE was dissolved in water (5 mg/mL) and 20 µL was diluted to a final volume of 800 µL, equivalent to 100 µg of extract. Then, 50 µL of 2 N Folin–Ciocȃlteu reagent (Merck Life Science, Milano, Italy) and 150 µL of 20% (*w*/*v*) Na_2_CO_3_ were added. After 30 min of incubation at 37 °C, samples were transferred to plastic cuvettes, and absorbance was measured with a Jasco V630 Spectrophotometer at a wavelength of 765 nm against a gallic acid calibration curve.

#### 4.4.2. Mass Spectrometry

Qualitative and quantitative analyses were carried out by LC–MS/MS using a LC system Surveyor MS PUMP PLUS (Thermo Fisher Scientific, Monza, Italy) coupled with an LTQ ion-trap mass spectrometer (Thermo Fisher Scientific, Monza, Italy) equipped with an ESI source operating in the negative mode. The column used was a XSelect HSS T3 XP (100 Å, 2.5 μm, 2.1 × 100 mm; Waters, Sesto San Giovanni, Italy) with a flow rate of 0.15 mL/min. A gradient elution was performed, and the mobile phase was a mixture of water containing 0.1% formic acid (A) and acetonitrile (B). The elution gradient was set as follows: 0–1 min (5% B), 1–10 min (5–100% B), 10–15 min (100% B), 15–25 min (100–5% B). The operating conditions for MS analysis were as follows: spray voltage, −5 kV; capillary temperature, 250 °C; sheath gas and auxiliary gas flow, 60 and 5 arbitrary units, respectively; tube lens, −110 V; total ion current (TIC); base peak-dependent mass range, *m/z* 230–1500; collision energy, 20 eV.

Compounds in the extract (injection: 25 μg) were identified by comparison of the retention times and mass spectra with those of authentic compounds (injection: 5 or 10 ng) commercially available at a high grade of purity.

### 4.5. Gene Expression Analysis

#### 4.5.1. RNA Extraction

At the end of the 6 h treatment, the medium was removed, and the cells were washed with PBS and lysed with QIAzol Lysis Reagent (QIAGEN GmbH, Hilden, Germany) according to the manufacturer’s instructions. The lysates were homogenized and kept frozen until the following RNA purification steps. Total RNA was isolated from cell lysates with miRNeasy Mini Kit (QIAGEN, Hilden, Germany), according to the manufacturer’s protocol. A set of RNase-free DNase (QIAGEN, Hilden, Germany) was used to provide efficient on-column digestion of genomic DNA. Total RNA was eluted with 35 μL of nuclease-free water. The concentration and quality of the purified RNA were assessed spectrophotometrically using a NanoDrop ND-1000 spectrophotometer (Thermo Fisher Scientific, Monza, Italy). Sample purity was estimated by measuring A260/280 and A260/230 ratios of spectrophotometric absorbance to check for possible copurified contaminants during the RNA isolation.

#### 4.5.2. cDNA Synthesis

Residual genomic DNA elimination and cDNA synthesis were performed with the RT^2^ First Strand Kit (QIAGEN, Hilden, Germany), according to the manufacturer’s indications, using 400 ng of total RNA for each sample.

#### 4.5.3. qPCR

The analysis of gene expression was performed using a 384-well PCR array related to human genes involved in wound healing (RT^2^ Profiler PCR Array PAHS-121Z Human Wound Healing; QIAGEN, Hilden, Germany). Each well of the array contained different primers for a specific target gene (in total 84 different target genes) or housekeeping gene for data normalization. Control wells were present to assess genomic DNA contamination, as well as the quality of the reverse transcription and PCR reactions. A quantity of cDNA equivalent to 400 ng of total RNA was mixed with SYBR^®^ Green Master Mix RT^2^ Reagent (QIAGEN, Hilden, Germany) according to the manufacturer’s instructions and aliquoted into the 384-well array. The real-time qPCR was performed using a CFX384™ Real-Time PCR Detection System (Bio-Rad Laboratories, Segrate, Italy). The threshold cycle value for each gene (C_t_) was automatically provided by software CFX Manager™ (Bio-Rad Laboratories, Segrate, Italy), depending on the amplification curves, and the threshold was manually set as recommended by the RT^2^ Profiler PCR Array manual. The C_t_ cut-off was set to 35. Expression data were normalized to the housekeeping gene RPLP0 (ribosomal protein lateral stalk subunit P0) and elaborated using the ΔΔC_t_ method based on Pfaffl equation [62].

### 4.6. NF-κB-Driven Transcription

HaCaT cells were transiently transfected with a reporter NF-κB-Luc plasmid (250 ng per well), containing the luciferase gene under the control of the E-selectin promoter characterized by three κB responsive elements, using Lipofectamine^®^ 3000 Transfection Reagent (Invitrogen^®^; Thermo Fisher Scientific, Monza, Italy). The plasmid NF-κB-Luc was a gift from Dr. N. Marx (Department of Internal Medicine-Cardiology, University of Ulm; Ulm, Germany). Sixteen hours later, cells were treated as previously stated. At the end of the treatment, the amount of luciferase produced into the cells was assessed using Britelite™ Plus reagent (Perkin Elmer, Milano, Italy) according to the manufacturer’s instructions. The luminescence deriving from the reaction between luciferase and luciferin was measured with a VICTOR X3 Multilabel Plate Reader (Perkin Elmer, Milano, Italy). The results (mean ± SD of at least three experiments) were expressed as percentage relative to stimulated control, which was arbitrarily assigned the value of 100%.

### 4.7. Measurement of IL-6, IL-8, IL-10, TSLP, and s-ICAM-1 Release

The release of IL-6, IL-8, IL-10, TSLP, and s-ICAM-1 was evaluated by enzyme-linked immunosorbent assay (ELISA) on culture media. The amount of intracellular IL-10 was assayed in whole-cell lysates obtained with a buffer containing 150 mM NaCl, 1% IGEPAL^©^ CA-630, and 50 mM Tris pH 8.0 supplemented with protease inhibitor cocktail (all from Merck Life Science, Milano, Italy). Sample loading was normalized according to the protein content, determined with the BCA method (Quantum Protein Assay Kit; Euroclone, Pero, Italy).

Human IL-6, IL-8, IL-10, TSLP, and ICAM-1 ELISA development kits were purchased from PeproTech (PeproTech, London, UK). Corning 96-well EIA/RIA plates (Merck Life Science, Milano, Italy) were coated overnight at room temperature with the capture antibody contained in the kit. The amounts of IL-6, IL-8, IL-10, TSLP, and s-ICAM-1 in the samples were detected by measuring the absorbance resulted from the colorimetric reaction between horseradish peroxidase enzyme and 2,2′-azino-bis(3-ethylbenzothiazoline-6-sulfonic acid) (ABTS) or 3,3′,5,5′-tetramethylbenzidine (TMB) substrate (Merck Life Science, Italy), according to the manufacturer’s instructions. The signal was read using a spectrophotometer (VICTOR X3; PerkinElmer, Milano, Italy) at 405 nm (ABTS) or 450 nm (TMB). IL-6, IL-8, IL-10, TSLP, and s-ICAM-1 levels were quantified through a standard curve supplied with the ELISA kit. The results (mean ± SD of at least three experiments) were expressed as percentage relative to stimulated control, which was arbitrarily assigned the value of 100%.

Preliminary time course experiments were performed to set the optimal experimental conditions for the subsequent experiments (see Appendix A).

### 4.8. Measurement of Intracellular cAMP Levels

After 9 days of differentiation, HaCaT cells were incubated for 24 h in presence of increasing concentrations of RNLE. At the end of the treatment, adherent cells were lysed with 100 µL/well of 0.1 M hydrochloric acid at RT for 20 min. Samples were collected using a scraper and subjected to centrifugation at 1000× *g* for 10 min. The supernatant was transferred into new tubes, and total protein content was evaluated by BCA method. Samples (50 µg of total proteins each) were diluted 1:2 with assay buffer and directly used for the evaluation of intracellular cAMP levels by a cyclic AMP ELISA kit (Cayman Chemical, Ann Arbor, MI, USA) according to the manufacturer’s protocol. Absorbance of samples was measured with a VICTOR X3 Multilabel Plate Reader (Perkin Elmer, Milano, Italy) at a wavelength of 405 nm.

### 4.9. Statistical Analysis

All data were expressed as the mean ± SD of at least three independent experiments. Gene expression results were calculated using the ∆∆C_t_ method. ∆∆C_t_ (log_2_ fold change) values were analyzed by multiple unpaired *t*-tests. ELISA assays were analyzed by unpaired one-way analysis of variance (ANOVA), followed by Bonferroni post hoc test.

Statistical analyses were performed using GraphPad Prism 8.0 software (GraphPad Software Inc., San Diego, CA, USA). Values of *p* < 0.05 were considered statistically significant.

## Figures and Tables

**Table 1:** Quantitative analysis of phenolic compounds occurring in RNLE.

Compound	Molecular Weight (*m/z* of [M − H]^−1^)	Retention Time (min)	wt.% in RNLE
Kaempferol aglycone	285	9.06	2.9%
Kaempferol-7-glucoside	447	7.53	3.8%
Kaempferol-3-glucoside	447	7.48	1.3%
Quercetin aglycone	301	8.52	1.9%
Hyperoside	463	7.26	0.8%
Rutin	610	7.09	1.6%
Chlorogenic acid	353	6.68	1.5%

## Data Availability

The data presented in this study are available on request from the corresponding author.
